# Differences in intestinal motility during different sleep stages based on long-term bowel sounds

**DOI:** 10.1186/s12938-023-01166-z

**Published:** 2023-11-02

**Authors:** Guojing Wang, Yibing Chen, Hongyun Liu, Xiaohua Yu, Yi Han, Weidong Wang, Hongyan Kang

**Affiliations:** 1 Key Laboratory of Biomechanics and Mechanobiology (Beihang University), Ministry of Education, Beijing Advanced Innovation Centre for Biomedical Engineering, School of Biological Science and Medical Engineering, Beihang University, Beijing, China; 2 Key Laboratory of Biomedical Engineering and Translational Medicine, Ministry of Industry and Information Technology, Chinese PLA General Hospital, Beijing, China; 3 Bioengineering Research Center, Medical Innovation Research Division, Chinese PLA General Hospital, Beijing, China; 4 Department of Pulmonary and Critical Care Medicine, Chinese PLA General Hospital, Beijing, China

**Keywords:** Sleep stages, The modified higher order statistics algorithm, Intestinal motility, Bowel sounds, Effective bowel sounds automatic recognition

## Abstract

**Background and objectives:**

This study focused on changes in intestinal motility during different sleep stages based on long-term bowel sounds.

**Methods:**

A modified higher order statistics algorithm was devised to identify the effective bowel sound segments. Next, characteristic values (CVs) were extracted from each bowel sound segment, which included 4 time-domain, 4 frequency-domain and 2 nonlinear CVs. The statistical analysis of these CVs corresponding to the different sleep stages could be used to evaluate the changes in intestinal motility during sleep.

**Results:**

A total of 6865.81 min of data were recorded from 14 participants, including both polysomnographic data and bowel sound data which were recorded simultaneously from each participant. The average accuracy, sensitivity and specificity of the modified higher order statistics detector were 96.46 ± 2.60%, 97.24 ± 2.99% and 94.13 ± 4.37%. In addition, 217088 segments of effective bowel sound corresponding to different sleep stages were identified using the modified detector. Most of the CVs were statistically different during different sleep stages ($p<0.05$). Furthermore, the bowel sounds were low in frequency based on frequency-domain CVs, high in energy based on time-domain CVs and low in complexity base on nonlinear CVs during deep sleep, which was consistent with the state of the EEG signals during deep sleep.

**Conclusions:**

The intestinal motility varies by different sleep stages based on long-term bowel sounds using the modified higher order statistics detector. The study indicates that the long-term bowel sounds can well reflect intestinal motility during sleep. This study also demonstrates that it is technically feasible to simultaneously record intestinal motility and sleep state throughout the night. This offers great potential for future studies investigating intestinal motility during sleep and related clinical applications.

## Introduction

In 2020, the journal *Cell* suggested that the primary cause of death during sleep was not the brain, not the heart, but the accumulation of reactive oxygen species in the intestines that caused the problem [1]. Other researchers found that about 68% of patients with functional dyspepsia also had sleep disorders [2].

The close relationship between gastrointestinal function and sleep as an important area for research, especially for a variety of diseases closely related to intestinal motility, such as inflammatory bowel disease, which have a common impact on sleep and affect quality of life [3]. In the 1990s, some qualitative and quantitative intestinal evaluating methods combined with polysomnography (PSG) recognized differences in intestinal motility during sleep in patients with intestinal disorders. However, the qualitative assessment methods based on subjective descriptions or questionnaires [4, 5] gave biased results and could not accurately assess the correlation between sleep stages and intestinal status [6]. In the following studies, some quantitative methods were used in combination with PSG to investigate the relationship between sleep and intestinal disease. The quantitative methods used in the studies included nasal cannula with pressure sensors indicating small bowel pressure [7–9], and abdominal surface electromyogram sensors detecting intestinal electrical activity [10].

Research on the effect of sleep itself on intestinal motility may go back to the early 1900s. Cannon described the differences in intestinal motility during waking and sleep through observational techniques [11]. Subsequently, the researchers quantified the effects of sleep on intestinal motility mainly through migrating motor complex (MMC), an intestinal contraction wave that begins in gastropathy through the colon. Some studies had concluded that sleep had significant inhibitory effect on gastrointestinal motility [12] and the cycles of MMCs and REM sleep were independent [13]. In recent years, quantitative evaluation using 3D-Transit capsules to monitor gastrointestinal motility [14] confirmed these MMC changes during sleep.

Among these quantitative methods, using a nasal cannula causes a strong sense of discomfort and a large load on the body [15, 16]; electromyograms have complex interference factors, so it is difficult to accurately evaluate intestinal electrical activity [17]; 3D-Transit capsules also require invasive measurement, which can cause a certain amount of discomfort. Moreover, the position of the capsule cannot be easily located and controlled [18]. There is also a risk of accidental expulsion of capsules throughout monitoring. Considering that subjects are nearly motionless during sleep, and the sleeping environment is tranquil, researchers have discovered that using the acquisition of bowel sounds (BSs) method to evaluate intestinal motility had obvious advantages [19]. Furthermore, BS monitoring fulfils the need for non-invasive and long-term monitoring during sleep.

The processing and analyzing methods of BS signals mainly performed de-noising processing on the original BS data, and then performed effective BSs segments (EBSs) recognition and classification after data conversion. The de-noising methods included the Wiener filter [20], wavelet denoising [21], adaptive filters [22], etc., to remove friction sounds, interference signals of the human body and other ambient noise. The recognition algorithm mainly used higher order statistics (HOS) [23, 24], fractal dimension [25, 26] and other methods to achieve data conversion, and then determined the thresholds to recognize EBSs. After 2015, researchers gradually applied artificial intelligence (AI) algorithms to BSs. Specifically, machine learning methods mainly include Bayesian classification [27], support vector machines [28], autoregressive sliding averaging [29], etc., and deep learning methods mainly include backpropagation neural network [30], convolutional neural network [31], hybrid convolutional and recurrent neural network [19], long–short-term memory [32], etc. Compared with conventional algorithms, AI algorithms are trained by a large amount of data to determine the parameters and models, and when the detection of large differences in the target, can be supplemented with training data and retrained, without the need to change the model can be extended to use, so it has a strong generalization ability [33]. In addition, the identification and classification of BSs can be calculated with accuracy varying from 75 to 95% [34]. However, the current AI algorithms mainly achieve relative long segments of BS classification [31, 32], which cannot achieve endpoint recognition. For the BSs acquired during sleep had relatively high quality because of the quiet environment and the static state of the participants. The HOS methods could totally achieve the accurate identification of EBSs. The main factors affecting the accuracy focused on the threshold. In previous studies, the threshold was either adjusted by varying the power of the overall back ground signals [24, or based on the histogram analysis of kurtosis with a fixed portion of 90% [29], or directly defined as the sum of the average and the standard deviation of the HOS values [35]. However, since BSs varied from person to person, which may be affected by the internal structure of the abdomen, age and gender, the fixed threshold used to identify EBSs of different participants would certainly affect the recognition accuracy.

In this study, we used our self-developed BS recorder to obtain long-term BSs. In addition, the HOS algorithms were adopted for EBSs recognition. Since the test environment did not change for each participant throughout the night, we selected 30 min of data for manual labeling. In addition, those labeled data were used to determine the threshold of each participant. The optimal threshold was then used to detect the EBSs throughout the night. After that, the time-domain, frequency-domain and nonlinear characteristic values (CVs) of EBSs within different sleep stages were extracted. Finally, intestinal motility was evaluated after statistical analysis of the CVs. This study takes the assessment of intestinal motility during sleep as the starting point, which not only enriches the physiological parameters of sleep-state assessment, but also provides new avenues for the diagnosis of intestinal motility diseases, which has important clinical and research significance.

## Results

The modified HOS algorithm (m-HOS) based on the annotation EBSs obtained the different optimal thresholds for each participant, as shown in Table 1.

**Table 1:** Evaluation parameters of the m-HOS algorithm and the optimal thresholds

Participants	$Accurary$(%)	$Sensitivity$(%)	$Specificity$(%)	Optimal thresholds
1	97.96	99.24	91.95	75.57
2	99.14	98.23	98.82	29.00
3	95.73	98.13	90.13	828.37
4	93.95	91.35	94.98	55.19
5	93.33	96.94	84.84	142.05
6	91.54	89.62	92.36	85.01
7	98.56	99.59	96.22	61.37
8	99.48	99.73	98.78	71.69
9	98.98	98.71	99.10	64.37
10	97.23	95.49	97.64	43.39
11	95.72	99.09	91.02	356.13
12	98.27	98.41	98.28	126.47
13	92.34	97.42	87.75	357.21
14	98.22	99.42	95.95	413.83
Mean (Stand deviation)	96.46 (2.60)	97.24 (2.99)	94.13 (4.37)	

Finally, we acquired a total of 6865.81 min of data from 14 participants. In addition, 217,088 segments of EBSs corresponding to different sleep stages were recognized, as shown in Table 2.

**Table 2:** Number of EBSs during different sleep stages

Sleep stages	Number of EBSs	Length of sleep time (min)
Awake	72672	1751.32
N1	16412	461.67
N2	67229	2493.32
N3	33858	1080.00
REM	26917	1079.50
Total	217088	6865.81

As these CVs of time, frequency and non-linear domains did not conform to the normal distribution, non-parametric tests were performed. Specifically, Kruskal–Wallis *H* tests were used to complete the analysis and the significance values were adjusted by the Bonferroni correction for multiple tests.

Table 3 shows the statistical analysis of time-domain CVS. The results in Table 4 show that the time-domain CVs were statistically different ($p<0.05$).

**Table 3:** Distribution ^a^ and statistical results of time-domain CVs

Time-domain CVs	Awake	N1	N2	N3	REM	$p$
$cv$	0.92 (0.38)	0.95 (0.38)	0.96 (0.37)	0.93 (0.37)	0.95 (0.36)	0.000
$E0$	31472.34(169325.93)	28228.17(179169.33)	21824.35(124121.42)	33160.73(194306.20)	24671.82(123788.59)	0.000
$Duration$	237 (416)	233 (358.75)	228 (161)	235 (163)	227 (142)	0.000
$Frequency$	0.63 (0.29)	0.57 (0.58)	0.48 (0.24)	0.45 (0.29)	0.30 (0.37)	0.004

^a^ Values are presented as median (interquartile range)

**Table 4:** Pairwise comparison results of time-domain CVs

Samples	$p$			
	$cv$	$E0$	$Duration$	$Frequency$
w-N1	0.000	0.000	0.000	1.000
w-N2	0.000	0.000	0.000	0.150
w-N3	0.014	0.261	0.000	0.115
w-REM	0.000	0.000	0.000	0.009
N1–N2	0.115	0.000	0.000	0.808
N1–N3	0.000	0.000	0.013	0.659
N1-REM	1.000	0.000	0.000	0.086
N2–N3	0.000	0.000	0.000	1.000
N2-REM	0.000	0.000	0.388	1.000
N3-REM	0.000	0.000	0.000	1.000

Table 4 shows the post hoc test results of the pairwise comparison of time-domain CVs. For $v$, the results indicated that the difference between two different stages were statistically significant except for N1–N2 and N1-REM. For$E0$, the results show that the difference between different stages were statistically significant except for w-N3. For$duration$, the results were that the differences between different sleep stages were significant except for N2-REM. For$frequency$, the results show that there were no significant differences between the groups except for w-REM.

**Fig. 1:**
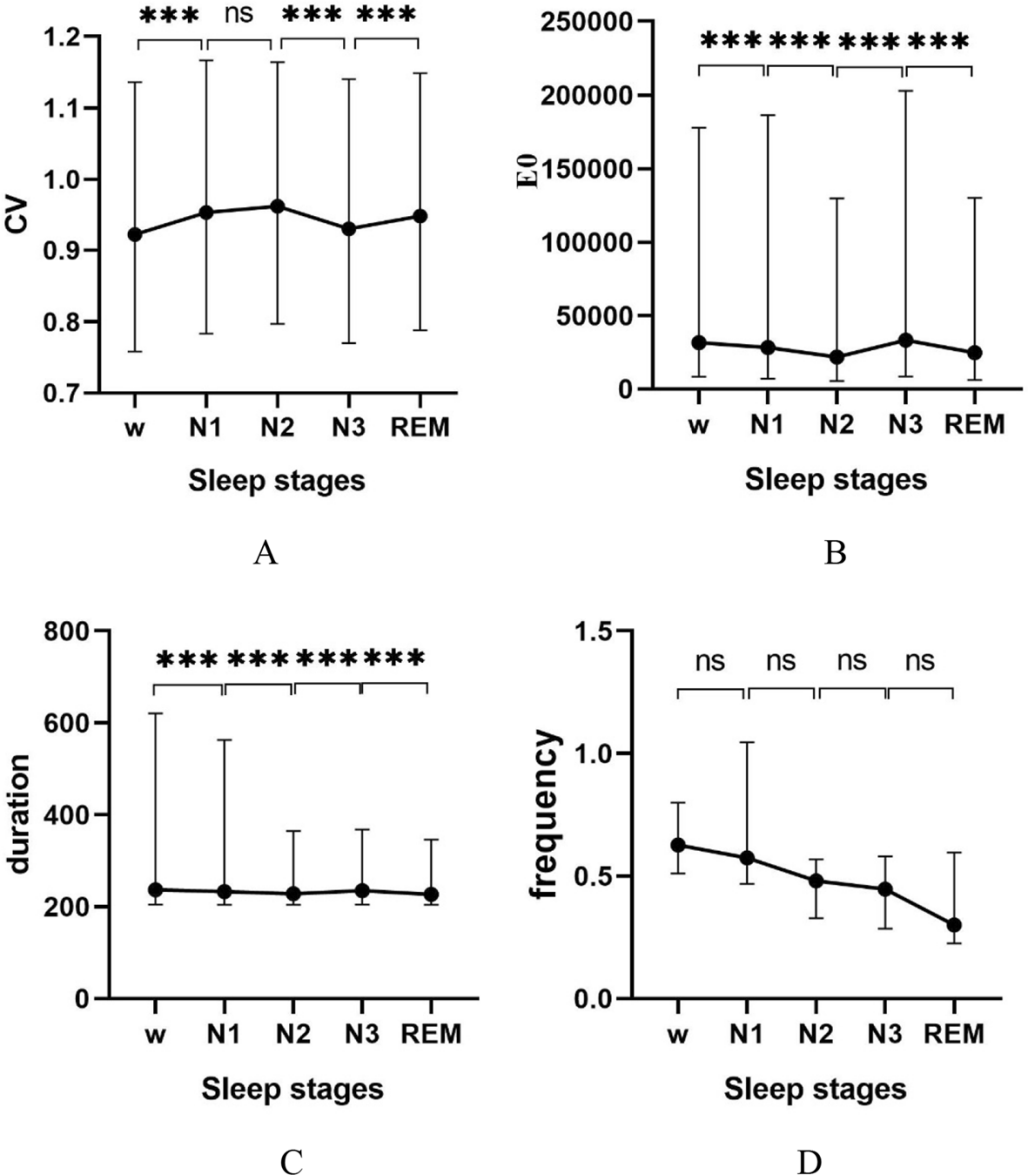
Distribution and trend of CVs in time domain. **A** Distribution of $cv$ expressed as median and quartile values. **B** Distribution of $E0$ expressed as median and quartile values. **C** Distribution of $duration$ expressed as median and quartile values. **D** Distribution of $frequency$ expressed as median and quartile values, where for ns, *p* > 0.05; *, *p* < 0.05; **, *p* < 0.01; ***, *p* < 0.001

Figure 1 shows the trend of CVs expressed as median and quartile values. Figure 1A shows $cv$ increased from w to N1 and N2, decreased to the minimum during N3, and then increased in REM. Figure 1B shows $E0$ deceased from w to N2, increased during N3, and decreased during REM. Figure 1C shows that the $duration$ decreased while entering sleep till N2, increased during N3, and decreased during REM. Figure 1D shows that $frequency$ almost did not change across sleep stages.

Table 5 shows the statistical analysis results of frequency-domain CVs. Table 5 shows the data distribution with the median and quartile values and the $p$ values were all less than 0.05, which meant that the difference for these four frequency-domain CVs during different sleep stages were statistically significant.

**Table 5:** Distribution^a^ and statistical results of frequency-domain CVs

Frequency-domain CVs	Awake	N1	N2	N3	REM	$p$
$FC$	140.93 (141.26)	141.15 (150.63)	156.46 (149.72)	129.14 (121.86)	144.17 (131.63)	0.000
${ER}_{5-300}$	0.90 (0.26)	0.90 (0.26)	0.88 (0.28)	0.93 (0.20)	0.91 (0.23)	0.000
${ER}_{300-500}$	0.06 (0.16)	0.06 (0.15)	0.07 (0.15)	0.05 (0.13)	0.06 (0.13)	0.000
${ER}_{500-1000}$	0.02 (0.07)	0.02 (0.08)	0.02 (0.09)	0.01 (0.04)	0.02 (0.07)	0.000

^a^Values are presented as median (interquartile range)

Table 6 shows the pairwise comparison results of frequency-domain CVs. For $FC$, the results show that the difference between any two different stages were statistically significant except for w-N1 and N1-REM. For ${ER}_{5-300}$, the post hoc test results showed that the difference between two different stages were statistically significant except for N1-REM. For ${ER}_{300-500}$, the results show that the different between two different sleep stages were statistically significant except between N1-REM. For ${ER}_{500-1000}$, the results show that the different between two different sleep stages were statistically significant except for w-N1, w-REM and N1-REM.

**Table 6:** Pairwise comparison results of frequency-domain CVs

Samples	$p$			
	$FC$	${ER}_{5-300}$	${ER}_{300-500}$	${ER}_{500-1000}$
w-N1	1.000	0.000	0.000	0.335
w-N2	0.000	0.000	0.000	0.000
w-N3	0.000	0.000	0.000	0.000
w-REM	0.000	0.000	0.000	1.000
N1–N2	0.000	0.000	0.000	0.000
N1–N3	0.000	0.000	0.004	0.000
N1-REM	0.890	1.000	1.000	0.255
N2–N3	0.000	0.000	0.000	0.000
N2-REM	0.000	0.000	0.000	0.000
N3-REM	0.000	0.000	0.030	0.000

**Fig. 2:**
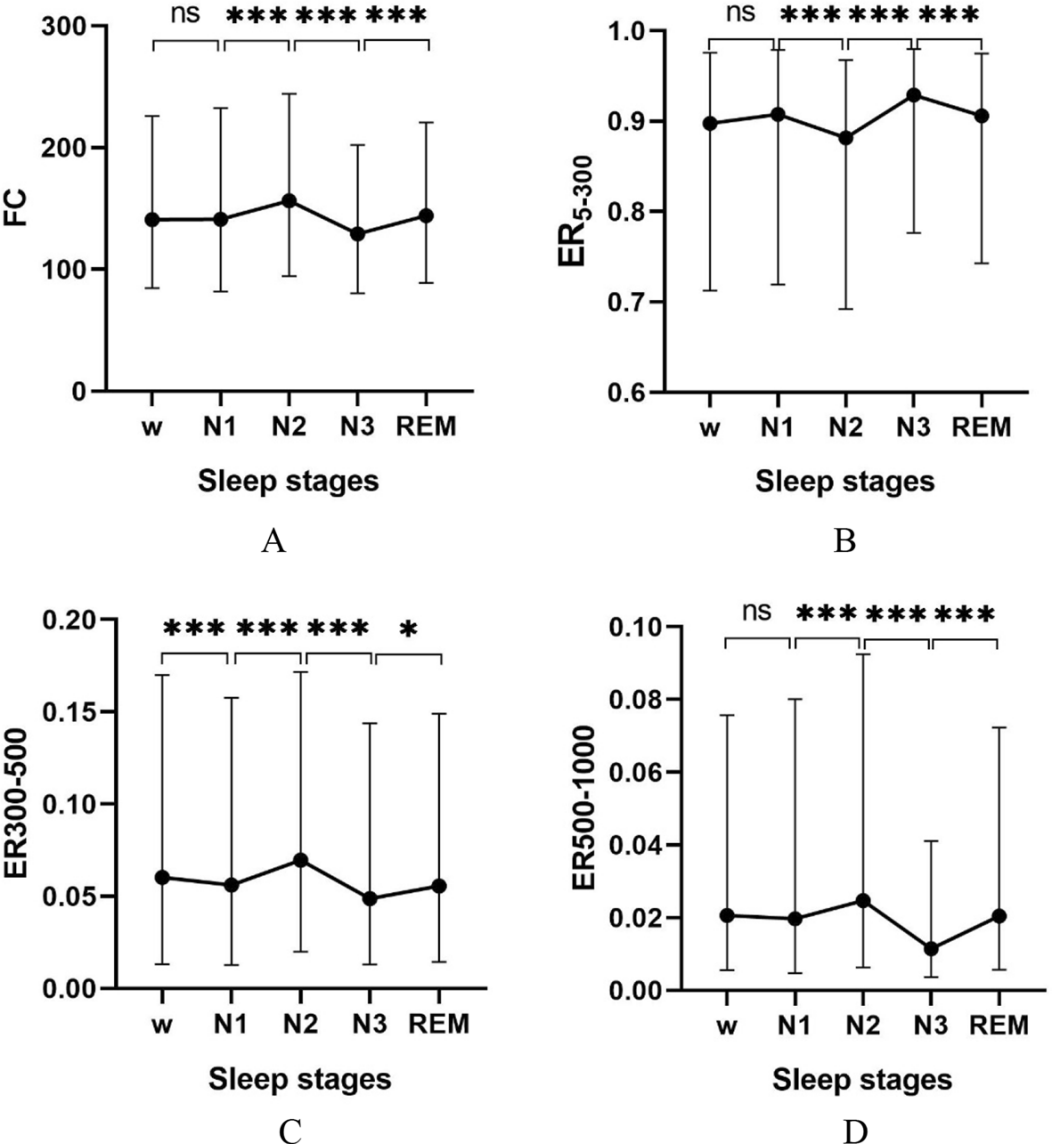
Distribution and trend of CVs in frequency domain. **A** Distribution of $FC$ expressed as median and quartile values. **B** Distribution of ${ER}_{5-300}$ expressed as median and quartile values. **C** Distribution of ${ER}_{300-500}$ expressed as median and quartile values. **D** Distribution of ${ER}_{500-1000}$ expressed as median and quartile values, where for ns, $p>0.05$; *, $p<0.05$; **, $p<0.01$; ***, $p<0.001$

Figure 2 shows the trend of frequency-domain CVs expressed as median and quartile values. Figure 2A shows that the trend of $FC$ increased from w to N1 and N2. It decreased while reaching N3 and increased in REM stage. The median and quartile values of $FC$ indicated that the frequency of BSs was mainly distributed below 300 Hz. Figure 2B shows that it deceased after entering sleep from w to N1 and N2, but it increased to highest in the deepest sleep state and decreased in REM. The median and quartile values of ${ER}_{5-300}$ indicated that the frequency distribution below 300 Hz accounted for more than 60%, which was consistent with the result of the above $FC$ distribution. Figure 2C shows that ${ER}_{300-500}$ increased from w to N1 and N2 and decreased in stage N3, and then increased in stage REM. The median and quartile values show that the ${ER}_{300-500}$ distribution was under 20%. Figure 2D shows that ${ER}_{500-1000}$ increased from w to N1 and N2, decreased in stage N3 and increased in stage REM. Furthermore, the median and quartile values show the ${ER}_{500-1000}$ distribution was under 15%.

The analysis of the frequency-domain CVs shows that the frequency range of BSs during sleep was mainly distributed below 300 Hz and the low-frequency components become larger as sleep gets deeper.

Table 7 shows the statistical analysis of nonlinear CVs. The results show that both the $p$ values were less than 0.05 which indicated that both the nonlinear CVs in different sleep stages were statistically different.

**Table 7:** Distribution^a^ and statistical results of nonlinear CVs

Nonlinear dynamic CVs	Awake	N1	N2	N3	REM	$p$
$FD$	1.0910 (0.0664)	1.0837 (0.0649)	1.0862 (0.0634)	1.0852 (0.0615)	1.0836 (0.0609)	0.000
$SampEn$	1.00 (0.31)	0.99 (0.29)	0.97 (0.28)	0.96 (0.29)	0.97 (0.29)	0.000

^a^Values are presented as median (interquartile range)

Table 8 shows the pairwise comparison results of nonlinear CVs. For $FD$, the post hoc test results show that the difference between two different stages were statistically significant except for N1-REM. For $SampEn$, the results show that the difference between stages were statistically significant except for N2-REM.

**Table 8:** Pairwise comparison results of nonlinear dynamic CVs

Samples	$p$	
	$FD$	$SampEn$
w-N1	0.000	0.000
w-N2	0.000	0.000
w-N3	0.000	0.000
w-REM	0.000	0.000
N1–N2	0.000	0.000
N1–N3	0.009	0.000
N1-REM	1.000	0.000
N2–N3	0.038	0.000
N2-REM	0.000	1.000
N3-REM	0.000	0.000

**Fig. 3:**
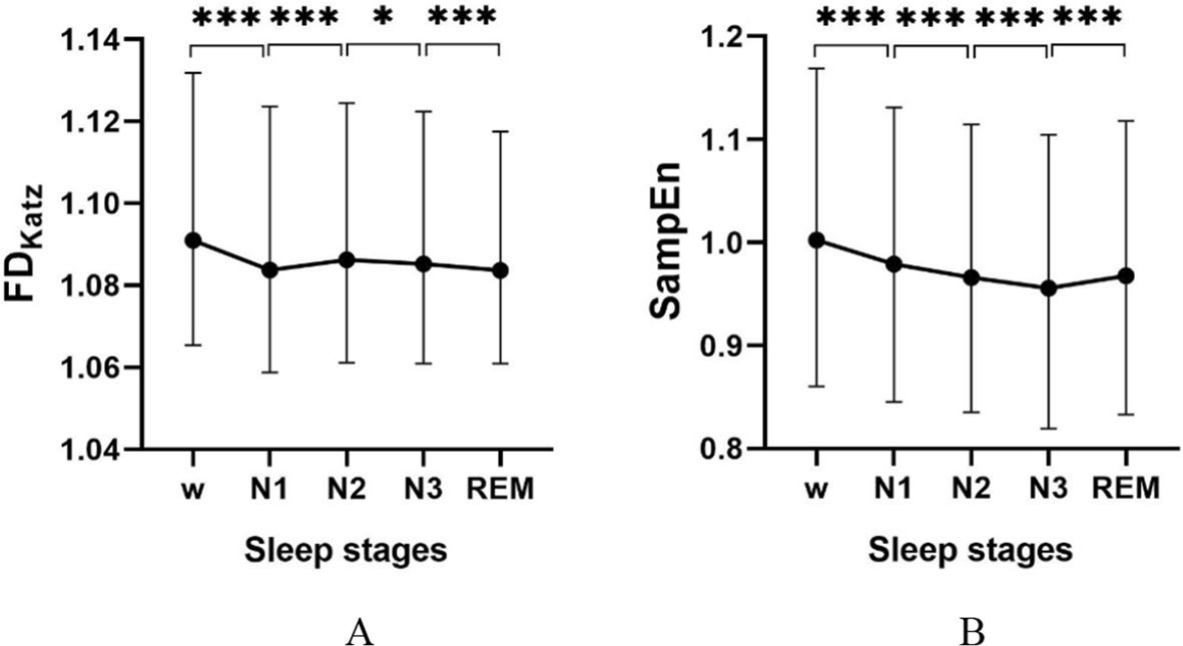
Distribution and trend of CVs in nonlinear domain. **A** Distribution of $FD$ expressed as median and quartile values. **B** Distribution of $SampEn$ expressed as median and quartile values, where for ns, $p>0.05$; *, $p<0.05$; **, $p<0.01$; ***, $p<0.001$

The trends of nonlinear CVs are shown in Fig. 3. Figure 3A shows that the trend of $FD$ decreased from w to N1, increased in stage N2, and decreased from N2 to REM. Figure 3B shows that the trend of $SampEn$ decreased after entering sleep reaching the lowest in stage N3 and increased in stage REM.

## Discussion

In our study, we proposed a m-HOS algorithm to find an optimal threshold for each participant, thus achieving relatively high accuracy in the identification of EBSs. Based on this, the CVs of EBSs were extracted to realize the quantitative assessment of intestinal dynamic during different sleep stages. This study demonstrates that it is technically feasible to simultaneously record intestinal motility and sleep state throughout the night. This offers great potential for future studies investigating intestinal motility during sleep and related clinical applications.

In the existing studies, although there were some studies on the evaluation of intestinal motility based on BSs, they mainly focused on the acquisition and recognition methods of EBSs [31, 36] and the correlation with intestinal diseases [37, 38], while only one study addressed bowel sounds during sleep [19]. However, this study only focused on the identification of bowel sounds at night and did not address the relationship between bowel motility and sleep.

In our study, PSG data and BS data were collected synchronously for the total duration of 6865.81 min. Using the m-HOS algorithm, 217088 segments of EBSs were automatically identified, and the average accuracy, sensitivity and specificity were 96.46 ± 2.60%, 97.24 ± 2.99% and 94.13 ± 4.37%. For each EBS, 4 time-domain CVs, 4 frequency-domain CVs and 2 nonlinear CVs were extracted, and then each CV was statistically analyzed for different sleep stages.

Furthermore, the overall trends of CVs were meaningful. In the time domain, as sleep deepened, EBSs fluctuated less along the mean value, became longer in duration time and did not differ in the number of occurrences per unit time, but the energy values of EBSs became significantly larger. In the frequency domain, the low-frequency component was the highest in stage N3, which was mainly concentrated below 300 Hz. In terms of nonlinear domain, fractal dimension and entropy both decreased from wake to sleep stages N1–N3 and increased during stage REM.

Surprisingly, the trends of the CVs in the above three domains are consistent with the EEG characteristics of different sleep stages. According to the standard criteria [39] of sleep stages annotation, the EEG of stage N3 is dominated by $\delta$ waves, and some other studies also showed that the EEG during stage N3 had high amplitude and low frequency [40, 41]. This coincides with the trends of BSs CVs in the time and frequency domains. In addition, for the nonlinear CVs of EEG in the previous review [39], a fractal component decreases from wake to sleep stages N1–N3 and increases during REM sleep, and from the entropy perspective, the complexity of sleep EEG also decreases from wake to sleep stages N1–N3 and increases during REM. Therefore, the trends of intestinal motility based on BSs in different sleep stages also coincide with the EEG signals during sleep from the entropy perspective. For the fractal dimension, the N3 period is also at a low value, but the REM has a slightly different trend. The changes of BS during different sleep stages were consistent with the changes of EEG, indicating the possible regulation of intestinal motility by the nervous system during sleep, which also provides a new idea to validate the theory of brain–gut axis [42, 43].

Finally, it was concluded that intestinal motility was statistically different during different sleep stages based on BSs, which indicates that intestinal motility does change with the sleep state under the regulation of the nervous system. This, in turn, suggests that BSs can be an effective way for long-term evaluation of intestinal motility.

Though our study obtained important results, there are also some limitations: (1) the EBSs recognition methods can be further optimized. For instance, the removal of the turning-over segments also removes the EBSs during these periods. Although this is a very small part of the total BSs during sleep and do not affect the overall results, it could be better. In addition, although the optimal threshold achieved an average accuracy of more than 90%, there are still some participants whose recognition performance is not particularly satisfactory. Follow-up studies consider incorporating artificial intelligence methods to further improve the generalization of the recognition methods. (2) the number of participants is not that large. There were 14 participants, but we obtained 217088 segments of EBSs throughout the night, which can provide relative sufficient data for the study of BSs during different sleep stages. In future studies, subjects could be further supplemented for more in-depth research. (3) it could be preliminarily concluded that BSs change with sleep stages. Further definitive patterns of changes in intestinal motility during sleep should be studied in depth in conjunction with sleep electroencephalography (EEG), which has been widely recognized to vary in different sleep stages, and it would be more convincing to study BSs in conjunction with sleep EEG. Thus, in future research, after increasing the number of subjects, we can combine the EEG signals to illustrate the characteristic changes of bowel sounds during different sleep stages. Furthermore, we can conduct correlation and coupling studies of EEG and bowel sounds during sleep, and explore the study of the brain–gut axis based on physiological signals.

## Conclusion

The m-HOS algorithm has high performance in identifying EBSs segments, with the average accuracy, sensitivity and specificity of 96.46 ± 2.60%, 97.24 ± 2.99% and 94.13 ± 4.37%, respectively. In addition, bases on the recognized EBSs and those CVs, we concluded that intestinal motility varies by sleep stages. Furthermore, the BSs are low in frequency, high in energy and low in complexity during deep sleep, which is consistent with the state of the EEG signal during deep sleep. In summary, the BSs can well express intestinal motility during sleep, which provides an effective method of assessing long-term intestinal motility, and this offers great potential for future studies investigating intestinal motility during sleep and related clinical applications.

## Materials and methods

### Participants and devices

The research was approved by the Medical Ethics Committee of the Chinese PLA General Hospital for clinical research (No. S2022-341-01). Participants with good sleep quality [44], defined as no insomnia, latency to sleep onset of less than 15 min, total sleep time of approximately 8 h, and number of awakenings after sleep onset of less than 2 were randomly recruited offline. Each participant signed an informed consent form. We also recorded some clinical factors that might influence intestinal motility, including age, gender and BMI [45]. The participants were confirmed the absence of intestinal disease to eliminate abnormal changes in BSs caused by gastrointestinal dysfunction. Participants were also instructed to eat little or no dinner and avoid medications and foods that affect sleep. The study was conducted in the Sleep Monitoring Center of the First Medical Center of the Chinese PLA General Hospital. Finally, 14 participants were recruited to complete one night of data collection, as shown in Table 9.

**Table 9:** Participant data

Age (years)	32.35 (9.30)^a^
Sex (M/F)	8/6
BMI*	23.72 (3.13)^a^
Sleep time (min)	490.41 (43.06)^a^

^*^Body mass index

^a^Values are presented as mean scores (standard deviation)

We used the BS recorder and the PSG device to acquire BS data and physiological signals during sleep simultaneously. The BS recorder, which was self-developed, had two channels based on the Knowles’ SiSonic MEMS microphone ((SPU1410LR5H-QB) with the port hole at the bottom. The channel for BSs (BS-channel) was to collect the raw BSs through the microphone chip on the front side of the circuit board (the port hole facing the human body), and the channel for noise sounds (NS-channel) was to collect the external ambient noise through the microphone chip on the back side of the circuit board (the port hole facing the outside). The MEMS microphone had a tightly matched sensitivity of ± 3 dB and an ultra-wide band flat frequency response of ± 2 dB in the 10–10 kHz frequency domain. The sound signals input to the 12 bits analog–digital converter (ADC) of MCU (STM32L151) which was chosen from STMicroelectronics. The sample rate was 8000 Hz. After processing of MCU, the data were stored on a Micro-SD card. In order for the device to adhere to the surface of the abdomen, we also designed the sticking splice and a buckle to fix the device.

PSG is an objective approach that is considered the gold standard for sleep measurement, especially for diagnosing physiologically based sleep disorders. A standard PSG montage was used following International 10–20 System guidelines [46]. The PSG device (EMBLA N7000) includes16-channel electroencephalography leads (EEG; F3, F4, C3, C4, O1, O2, M1, M2, GRD/REF), electromyography (EMG; 3 sub-mental leads), and electrocardiography (ECG; R/L Arm). Participants also wore thoracic and abdominal respiratory belts to monitor respiration.

Before the test, the technician wore the PSG sensors as requested and debugged them to ensure that each channel worked normally. The BS recorder was adhered on the surface of the right lower abdomen.

Synchronization of the two devices was achieved by creating a synchronization signal by tapping the sensors against each other. Specifically, PSG was selected to use the leg myoelectric sensor, which can form a pulse signal for the tapping, and the pickup port of the BS recorder was used to tap with the leg myoelectric electrodes to form a synchronization signal for the two independent devices, which in turn achieves synchronization of the devices.

#### Data annotation

In our study, data annotation included the EBSs and sleep stages annotation. EBSs annotation was achieved by two experienced clinicians in a double-blind manner. 30 min of bowel sound data from each participant’s entire night were selected for labelling. The reason for selecting a participant's 30-min BSs was to reduce the manual annotation workload compared to the full night’s data and, by manually annotating the local segment data, to set different HOS thresholds for each subject to improve the generalization ability of the effective bowel sound detection algorithm. While the HOS could assess the non-Gaussianity of the signal, the main factors affecting the non-Gaussianity of the bowel sound signal were the differences in the internal structure of the abdominal intestinal tract in different subjects, which mainly included the thickness of the abdominal intestinal wall, the distance between the intestinal tract and the surface of the abdomen, the thickness of the abdominal fat, and so on. In addition, the 30 min could reflect the whole night state as the test environment remained virtually unchanged throughout the night. Furthermore, to ensure consistency in the selection of labelled segments across subjects, we chose a period during each subject’s stage N3 as the labelled segment. As stage N3 generally lasted 20–40 min [47], 30 min was chosen as the labelled segment.

The specific labelling rule was that the EBSs were longer than 10 ms and two EBSs with an interval of less than 100 ms were regarded as one EBS. The EBSs labeled by both clinicians would be the final EBSs, otherwise they would be abandoned.

The sleep stages were labeled in 30-s epochs in compliance with standard criteria [48] by an experienced technician who had passed the Registered Polysomnographic Technologist (RPSGT) examination. The sleep stages included an awake stage (w), a transition stage from wakefulness to sleep (N1), a light sleep stage (N2), a deep sleep stage (N3) and a rapid eye movement sleep stage (REM).

### EBSs recognition algorithm

**Fig. 4:**
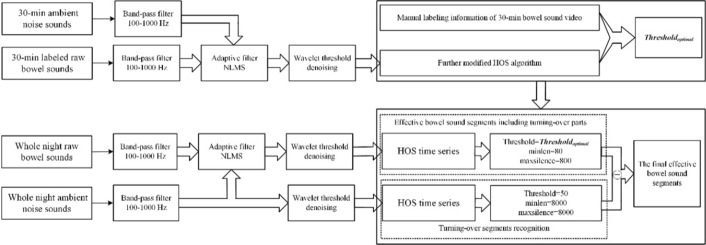
Flow chart of BS data processing and EBSs recognition. The optimal threshold was determined based on the labeled 30-min BS audio of each participant. Then, the whole night’s EBSs for each participant would be identified by the automatic recognition algorithm with the optimal threshold

In our study, the EBSs recognition algorithm first determined the optimal threshold based on the annotated 30-min BS audio of each participant. Then, with the optimal threshold, the recognition algorithm could be used to identify the whole night’s EBSs. Figure 4 shows the flow chart of EBSs recognition.

#### Data denoising

The noise of bowel sounds during sleep mainly includes heart sounds, background noise and noise caused by turning over. In this section, the processing of heart sounds and background noise is mainly implemented, and the noise caused by turning over is eliminated after subsequent identification. The denoising methods included the bandpass filtering, adaptive filtering and wavelet threshold denoising.

The bandpass filter filtered out the noise outside the frequency band of BSs. Some existing research described that the BS signals were mainly distributed in the range of 100–500 Hz [49, 50], 5–600 Hz [29], and 100–1000 Hz (mostly 100–800 Hz)[36]. To ensure the retention frequency range of BSs and the removal of the main noise, we selected the bandpass filter of 100–1000 Hz. More importantly, such a bandpass filter filters out the influence of the first and second heart sounds on the bowel sounds [51].

**Fig. 5:**
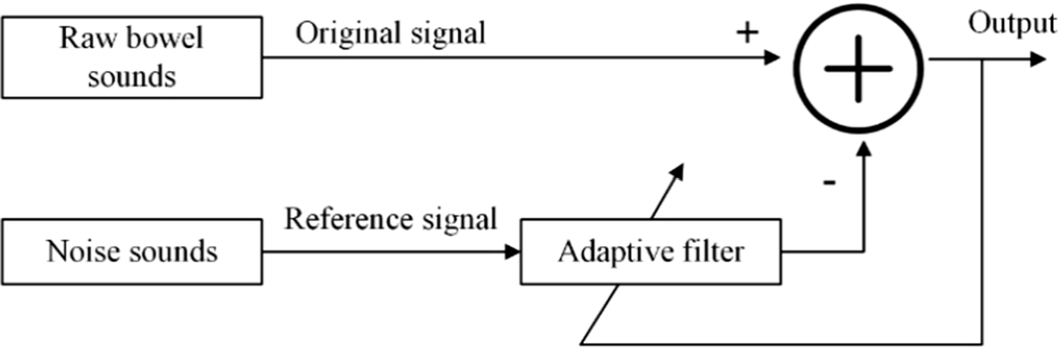
Adaptive filter architecture

The adaptive filter accomplished the denoising of ambient noise based on the two-channel configuration, as shown in Fig. 5. In this study, we chose NLMS [52] to achieve the adaptive noise canceller (ANC) [53].The parameters that had to be set for NLMS included the filter order and the step size [54], and the parameters that measured the performance of the filter were mainly MSE, SNR and correlation coefficient [53, 55].The filter order affected the computation time of the filter, the larger the filter order, the longer the computation time required; for the step size setting, the MSE decreased when the step size was small, and the convergence speed was accelerated when the step size was large. We considered the computation time, convergence speed and filtering effect simultaneously and set the filter length to 64 and the step size to 0.001 [53]. To evaluate the filtering effect, we mainly used the parametric correlation coefficient. Since the experimental procedure was performed in the same monitoring environment for all subjects, the ambient noise was kept essentially constant, which happened to be the reference input of the filter, and the output of the filter was the estimate of the ambient noise in the raw bowel sound signal, and the correlation coefficient was the correlation between the ambient noise and the estimate of the ambient noise. We performed adaptive filtering on 10 randomly selected 10 s segments of data from each of the 14 subjects to ensure that the correlation coefficient was greater than 0.9 to verify that the filter parameters were set to ensure that the filter had good filtering performance.

**Fig. 6:**
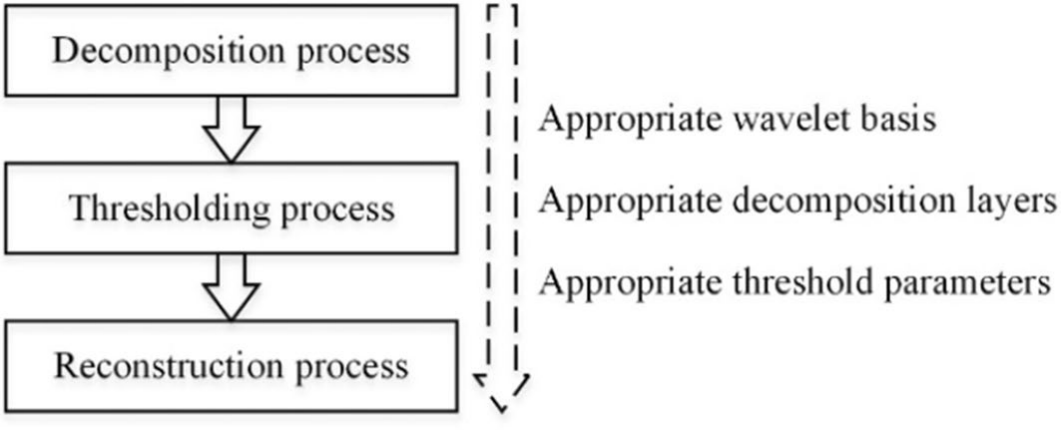
Procedure and parameters for wavelet threshold denoising

Wavelet threshold denoising (WTD) was used to suppress the noise component concentrated in the detailed component of wavelet decomposition using the *Mallat* pyramidal algorithm. The WTD process included decomposition processes, thresholding processes and reconstruction processes, as shown in Fig. 6. According to the comparison of the filtering effect, the *sym6* wavelet basis was selected and the number of decomposition layers was determined as 6. In addition, the threshold was calculated using the *Birge–Massart* algorithm for the threshold denoising [56].

#### Determination of optimal thresholds based on the 30-min labeled BSs

**Fig. 7:**
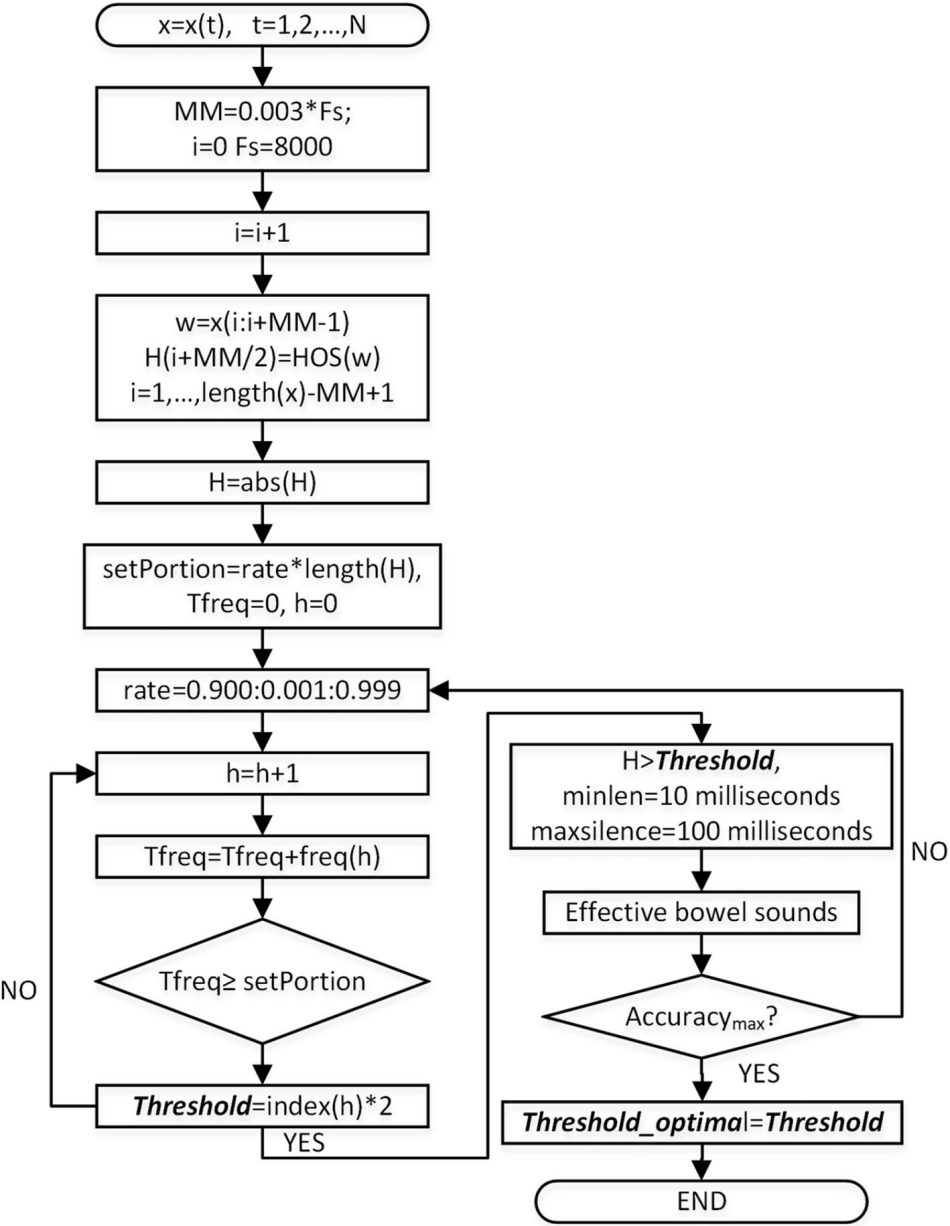
Flow chart of m-HOS algorithm

After data denoising, EBSs recognition was carried out. In the previous study [29], the modified iterative kurtosis-based detector (IKD) was used for the separation of the EBSs based on the kurtosis of a sliding-window BSs and a histogram analysis of the kurtosis time series (K). The percentage of the total frequency of K was fixed as 90% based on experience which may affect the recognition accuracy. Therefore, we devised the m-HOS based on the annotation EBSs. Specifically, for the 30-min labeled BSs of each participant, the histogram analysis of the HOS time series was obtained, and then the percentage was dynamically adjusted from 90 to 100% to determine the optimal threshold until the best detection performance was obtained based on the labelled EBSs. Figure 7 shows the flow chart of the m-HOS algorithm.

### Obtaining the HOS time series

The 30-min BSs were labeled manually. The sliding window was $MM=0.003\times Fs$, where $Fs$ was the sampling rate, and the constant 0.003 was set based on experience. The HOS time series (H) was calculated within the sliding window. In our study, the third-order-statistics cumulant of the BSs was calculated using the *cum3est* function [57] in the MATLAB software.

### Determining the optimal threshold for each participant

The temporary threshold was used for the EBSs recognition from the raw BSs. First, the histogram of H and the frequencies (*freq*) was calculated. Then, the sum of the *freq* reached the *setPortion* of the total frequencies and the temporary threshold was set as twice the recent index. The final optimal threshold was adjusted with the *setPortion* from 0.900 to 0.999.

With the temporary threshold, the corresponding EBSs were recognized from H. The target EBSs whose corresponding H values should be larger than the temporary threshold. In addition, the recognized rules were be consistent with the labeling rules.

The EBSs detected with the temporary threshold were compared with the manually labeled EBSs to evaluate the performance. Specifically, we used the $Accurary$ to evaluate the recognition performance with different temporary thresholds. While the $Accuracy$ achieved the maximum value, the corresponding temporary threshold was the optimal threshold for the participant. The $Sensitivity$ and $Specificity$ could help to reflect recognition performance.

**Fig. 8:**
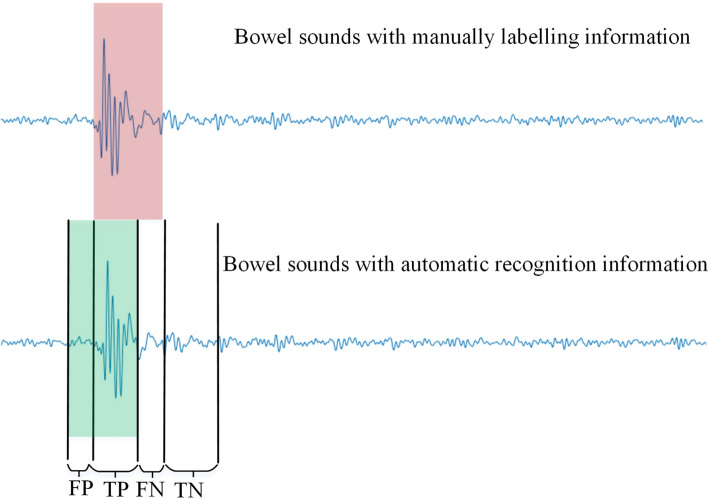
Evaluation parameters definition of the m-HOS algorithm

Figure 8 shows the definition of evaluation parameters. The red part was the manually labeled EBS, and the green part was the detected EBS. Several parameters were defined as follows: TP (the annotated EBS was correctly recognized as a true EBS), FP (the non-annotated segment was incorrectly identified as EBS), TN (the non-annotated segment was correctly identified as the noise part), FN (the non-annotated segment was incorrectly recognized as the noise part). The $Accurary$, $Sensitivity$ and $Specificity$ were defined as Eqs. (1), (2) and (3):

1 $$Accuracy=\frac{{E}_{TP}+{E}_{TN}}{{E}_{TP}+{E}_{FP}+{E}_{FN}+{E}_{TN}}$$

2 $$Sensitivity=\frac{{E}_{TP}}{{E}_{TP}+{E}_{FN}}$$

3 $$Specificity=\frac{{E}_{TN}}{{E}_{FP}+{E}_{TN}}$$

where ${E}_{(.)}$ denoted the energy of the corresponding segment. The reason for using the energy to calculate the evaluation parameters was that it could better express the recognition effect of transient signals with sudden energy aggregation-like EBSs. On one hand, it could avoid the recognition bias caused by the artificially labeled boundaries, and on the other hand, for the long-term BSs recognition, some particularly low-energy EBSs that were not recognized had little effect on the whole night BSs.

#### EBSs recognition and CVs extraction of the whole-night’s data

The optimal threshold of each participant was used for the EBSs identification of the whole night. The same as the EBSs recognition of labeled 30-min BSs, the HOS time series of the whole-night BSs after denoising were obtained. Then, the determined optimal thresholds of different participants, combined with the labeling rules, were applied to the EBSs recognition of the whole-night’s BSs.

### The recognition and removal of turning-over segments

**Fig. 9:**
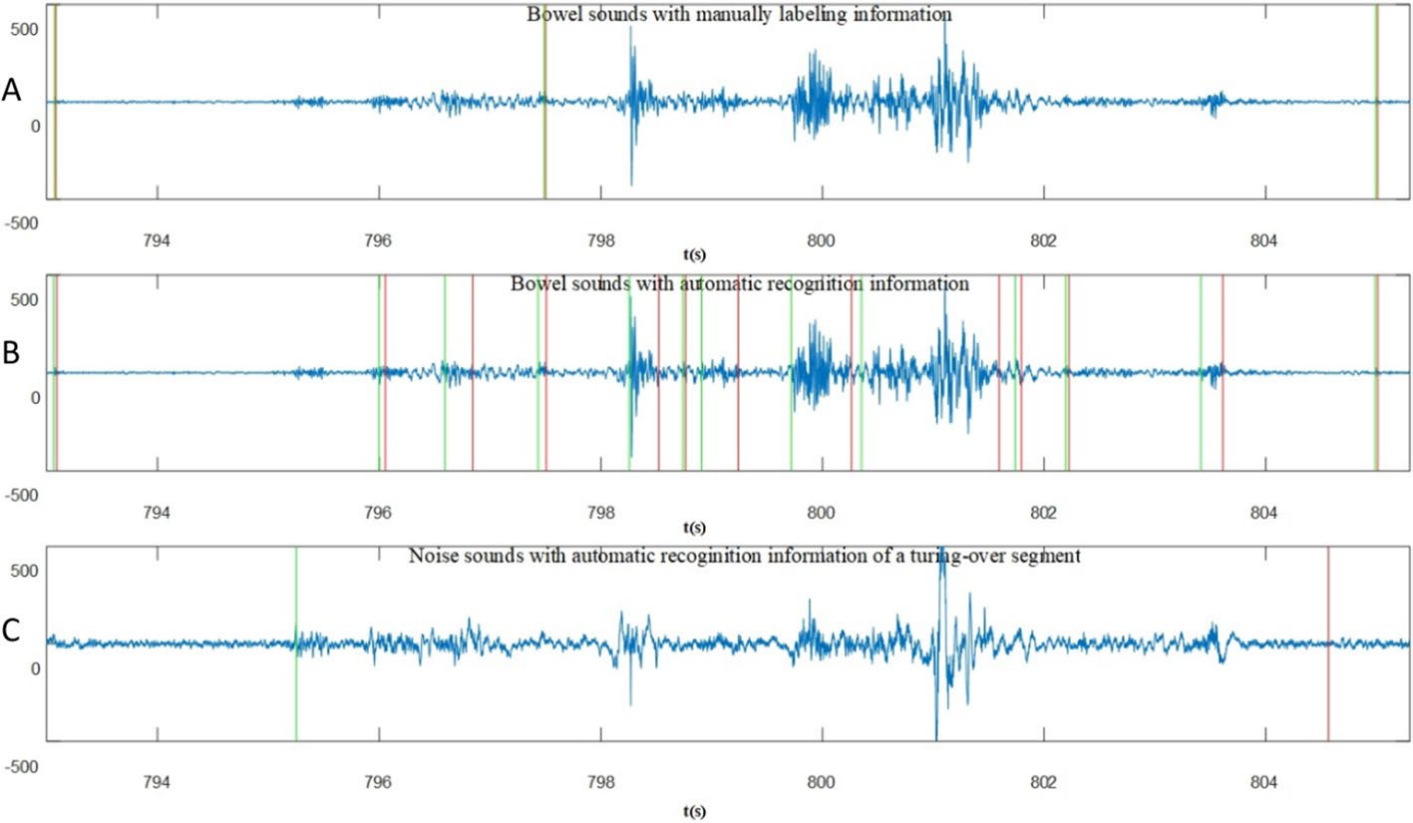
Turning-over segment recognition:** A** BSs with manually labeling information of EBSs. **B** BSs with automatic recognition information of EBSs. **C** noise sounds with automatic recognition information of a turning-over segment. Green lines: the start points of the segments. Red lines: the end points of the segments

Before the whole night’s EBSs recognition, we also recognized the turning-over segments for further improving the recognition performance. Specifically, we used the NS-channel data to recognize the turning-over segments. First, we calculated the HOS time series of the denoised NS-channel data and the length of sliding window was still $MM=0.003\times Fs$. Because the turning-over segments were evident and were a small amount, the parameters were directly determined by experience. The threshold was set at 50, the minimum length of turning-over segments at 1 s and the maximum length of interval between two turning-over segments at 1 s. Figure 9 shows one example of turning-over segment recognition. After the turning-over segments were recognized, the EBSs, while the turning-over segments happened were discarded.

### CVs extraction of EBSs during different sleep stages

In this study, CVs of time domain, frequency domain and nonlinear dynamics were extracted which described the intestinal states from various angles.

The time-domain CVs involved $cv$, $E0$, $duration$, and $frequency$. $cv$ was the coefficient of variation indicating the fluctuation of data. $E0$ was the energy of each EBS. $duration$ was the length of each EBS indicating the duration time. $frequency$ was the number of EBSs during a corresponding sleep stage.

The frequency-domain CVs involved $FC$, ${ER}_{5-300}$,${ER}_{300-500}$,${ER}_{500-1000}$. $FC$ was the centroid frequency describing the frequency with large components in the power spectrum. ${ER}_{5-300}$, ${ER}_{300-500}$ and ${ER}_{500-1000}$ represented the power in the 5–300 Hz, 300–500 Hz and 500–1000 Hz to the total power, respectively.

The nonlinear CVs included fractal dimension ($FD$) and sample entropy ($SampEn$) which were based on the concepts of fractals and entropy like for EEG signals during sleep [41. $FD$ was calculated by Katz algorithm based on the box-counting dimension, which could measure the unevenness and complexity of signals. $FD$ was defined as the following equation:

4 $$FD=\frac{{\mathrm{log}}_{10}(L)}{{\mathrm{log}}_{10}(d)}$$

where $L$ was the total length of the time series and $d$ was the estimated diameter, which was regarded as the distance between the first point of the series and the farthest point of the series.

Entropy values reflect the number of times the patterns in a signal are repeated and thus measure the randomness and predictability of stochastic process and in more general terms, increase with greater randomness [43]. In our study, the sample entropy was chosen to express entropy. Compared with approximate entropy, the sample entropy did not include the comparison with its own data segment when calculating the approximation, so the calculation error was small and did not depend on the data length, which was suitable for EBSs of different lengths. The sample entropy could be computed as Eqs. (5) and (6):

5 $$SampEn\left(m,r,N\right)=-\mathrm{ln}[\frac{{U}^{m+1}(r)}{{U}^{m}(r)}]$$

6 $${U}^{m}\left(r\right)={[N-m\tau ]}^{-1}\sum_{i=1}^{N-m\tau }{C}_{i}^{m}(r)$$

where $N$ was the length of a time series, $m$ was the pattern length, $r$ was the tolerance value, and $\tau$ is the time delay. ${C}_{i}^{m}\left(r\right)$ is defined as the following equation:

7 $${C}_{i}^{m}\left(r\right)=\frac{{B}_{i}}{N-(m+1)\tau }$$

where $${B}_{i}=number \,of\, j\, where d\left|{X}_{i},{X}_{j}\right|\le r$$.

After the CVs extraction of all the EBSs, we classified the CVs corresponding to different sleep stages according to the timepoint, where the EBSs were located.

### Statistical analysis

Statistical analyses were completed for CVs of the five sleep stages using IBM SPSS Statistics 25. Before statistical analysis, a normal distribution test was performed using the Kolmogorov–Smirnov test. For data satisfying the normal distribution, the homogeneity of variance test should be performed. The value of $p<0.05$ was considered to indicate statistical significance and the trend of different CVs during different sleep stages was expressed by the mean scores plot. For data not satisfying normal distribution, nonparametric tests should be performed. Similarly, $p<0.05$ indicated statistical significance and the data distribution could be expressed as median and quartile values.

## Data Availability

The data sets generated during and/or analyzed during the current study are available from the corresponding author at reasonable request.

## Abbreviations

CVs: Characteristic values
MMC: Migrating motor complex
PSG: Polysomnography
BS: Bowel sound
EBSs: Effective BSs segments
HOS: Higher order statistics
AI: Artificial intelligence
CVs: Characteristic values
m-HOS: Modified HOS algorithm
ADC: Analog–digital converter
ANC: Adaptive noise canceller
WTD: Wavelet threshold denoising
IKD: Iterative kurtosis-based detector
